# The risk of diabetes after giving birth to a macrosomic infant: data from the NHANES cohort

**DOI:** 10.1186/s40748-021-00132-8

**Published:** 2021-05-12

**Authors:** Corrie Miller, Eunjung Lim

**Affiliations:** 1 Department of Obstetrics, Gynecology and Women’s Health, John A. Burns School of Medicine, University of Hawaii, Honolulu, USA; 2 Department of Quantitative Health Sciences, John A. Burns School of Medicine, University of Hawaii, Honolulu, USA

**Keywords:** Diabetes screening, Macrosomia, Diabetes mellitus, Gestational diabetes, Large for gestational age

## Abstract

**Aims:**

Gestational diabetes (GDM) increases the risk of developing type 2 diabetes and thus warrants earlier and more frequent screening. Women who give birth to a macrosomic infant, as defined as a birthweight greater than 9 lbs. (or approximately 4000 g), are encouraged to also get early type 2 diabetes screening, as macrosomia may be a surrogate marker for GDM. This study investigates whether a macrosomic infant, as defined as 9lbs, apart from GDM, increases the risk for diabetes later in life.

**Methods:**

Data on parous women from the National Health and Nutrition Examination Survey (NHANES) 2007–2016 were utilized. Rates of diabetes were compared in those with and without macrosomic infants in Rao-Scott’s chi-square test. Multiple logistic regression was used to test the independent effect of macrosomia on type 2 diabetes controlling for the confounding covariates and adjusting for the complex sampling design. To investigate how onset time affects diabetes, we implemented Cox proportional hazard regressions on time to have diabetes.

**Results:**

Among 10,089 parous women, macrosomia significantly increased the risk of maternal diabetes later in life in the chi-square test and logistic regression. Independent of GDM, women who deliver a macrosomic infant have a 20% higher chance of developing diabetes compared to women who did not. The expected hazards of having type 2 diabetes is 1.66 times higher in a woman with macrosomic infant compared to counterparts.

**Conclusions:**

Women who gave birth to a macrosomic infant in the absence of GDM should be offered earlier and more frequent screening for type 2 diabetes.

## Introduction

Early screening for Type 2 diabetes and impaired glucose tolerance in the adult patient is imperative to prevent longstanding complications of the condition. Identifying risk factors such as family history, lifestyle factors or obstetric complications may prompt healthcare providers to perform earlier and more frequent screening for type 2 diabetes [1]. A pregnancy affected by gestational diabetes (GDM) is one such risk factor that increases the chance of developing type 2 diabetes later in life. Approximately one-third of women who had GDM will have impaired glucose metabolism 6–12 weeks after giving birth, and between 15 and 70% will develop type 2 diabetes in the future [2, 3]. Overall, studies consistently show that women with a history of GDM have a sevenfold increased risk of developing type 2 diabetes compared with women without the diagnosis [4, 5]. Thus, women are encouraged to get screened every 1 to 3 years for diabetes after giving birth if their pregnancy was affected by GDM [6].

Women who are unable to control GDM may give birth to large for gestational age (LGA) infants due persistent maternal hyperglycemia or a birthweight in the 90th percentile for gestational age. This is preceded by fetal macrosomia in utero, which refers to excessive fetal growth between 4000 and 4500 g regardless of gestational age [7]. Data from the National Center for Health Statistics show that 8% of all live-born infants in the United States weigh 4000 g or more [8], and not all neonates with increased birth weight result from GDM affected pregnancies. Other risk factors include maternal obesity, excessive gestational weight gain, a prior macrosomic infant, post-term pregnancy, increasing maternal age [9], and hyperlipidemia [2, 10–12]. In a large retrospective cohort study of nearly 10,000 women, the rate of LGA newborns without GDM ranged from 7.7% in normal-weight women to 12.7% in obese women, compared to 13.6% in normal-weight women and 22.3% in obese women affected by GDM [13]. In this particular cohort, among women without GDM, 21.6% of LGA infants were attributable to increased maternal body mass index (BMI).

There are significant risks of macrosomia for both mother and fetus. Maternal risks include postpartum hemorrhage, increased risk of cesarean delivery and third and fourth-degree vaginal lacerations. Fetal risks include shoulder dystocia with nerve injury, possible asphyxia, low 5-min APGAR scores, prolonged ventilation, stillbirth and infant mortality [14]. Later in life, there is known to be epigenetic imprinting on the fetus, potentiating risks of metabolic syndrome and glucose intolerance for the child [11, 15, 16]. Control of maternal hyperglycemia and maternal gestational weight gain during pregnancy are the most important preventative measures in avoiding fetal macrosomia [17]. However, elevated levels of insulin in cord blood is seen in macrosomic infants born to non-diabetic mothers [18], demonstrating that outside of preexisting maternal diabetes and uncontrolled GDM, other risk factors for delivery of a macrosomic infant exist.

The American Association of Clinical Endocrinologists (AACE) [19] and the American Diabetes Association (ADA) [20] recommend screening women for type 2 diabetes if they have given birth to a 9 lb. infant, as this is thought to represent impaired glucose tolerance and be a surrogate for undiagnosed GDM. Yet due to the multifactorial etiology of increased birth weight, this may not always be an accurate marker for impaired glucose tolerance. Kew et al. looked at glucose intolerance 3 months postpartum and did not find an association with LGA birthweight in the absence of diabetes [21]. Other studies with longer-term follow up for development of type 2 diabetes in women with macrosomic infants is limited.

To fill this gap, this study aims to discover whether a macrosomic infant alone is a marker for developing type 2 diabetes later in life, with the theory that fetal macrosomia may be a precursor to the development of impaired glucose tolerance beyond normal pregnancy physiology. We hypothesize that macrosomia increases the risk of diabetes later in life, even after adjusting for GDM and sociodemographic factors.

## Methods

### Data source

The population for this study was chosen from the National Health and Nutrition Examination Survey (NHANES) database from survey years 2007 to 2016 [22]. The NHANES sample represents the total noninstitutionalized civilian U.S. population residing in the 50 states and District of Columbia. The NHANES sampling design is a four-stage sampling design, starting with Primary Sampling Units from all U.S. counties; second stage consists of census blocks, the third of dwelling units, and finally persons within those households. A subsample of individuals was selected from all eligible members within a household based on sex, age, race and Hispanic origin, and income. The subsampling rates and designation of potential sampled participants within screened households were arranged to provide approximately self-weighting samples for each subdomain and to maximize the average number of sampled participants per sample household. Hispanic origin individuals were oversampled to produce the desired number of sampled participants in the difficult-to-recruit domain [23]. The NHANES created weights accounting for this complex survey design including oversampling, survey non-response, and post-stratification adjustment to match U.S. population [24].

### Variable selection

The primary outcome was a diagnosis of diabetes. This was coded as “Yes” if there was self-reported diagnosis by their doctor that they had diabetes mellitus, if they were currently taking insulin or diabetes pills, or had diagnostic laboratory criteria from the NHANES laboratory data (i.e., hemoglobin A1C > 6.5% or fasting blood glucose > 125 mg/dL).

The independent variable of macrosomia was defined as the positive answer in the question – “Did you have an infant weighing >9lbs?” The sociodemographic variables included in this study were race/ethnicity, age, and obesity. Race/ethnicity was categorized as White, Black, Mexican Hispanic, Other Hispanic and Other race including multi-races. Age was categorized into 20–44, 45–64, and 65 years or older. Obesity was defined using body mass index (BMI): underweight or normal (BMI < 25), overweight (25 ≤ BMI < 30), and obese (BMI ≥ 30). Reproductive confounding variables were obtained from the “Reproductive Questionnaire,” during the same cycle years, including history of GDM, age at time of GDM diagnosis, age at time of delivering an macrosomic infant, and parity (categorized into primiparous and multiparous). All participants who had a diagnosis of diabetes at a younger age of than the time of having an macrosomic infant and those that were never pregnant were excluded.

### Statistical analysis

Data were summarized using frequencies with weighted percentages and weighted means with standard errors. Demographic and potential confounding variables were compared in bivariate analyses to the exposure of macrosomia via Rao-Scott chi-squared tests for categorical variables and two sample t-test for continuous variables. The variables were also compared among those with and without the primary outcome of diabetes in univariable logistic regression analyses. The primary outcome of diabetes mellitus was evaluated in a multiple logistic regression analysis with all covariates chosen. To investigate how onset time affects diabetes, we implemented Cox proportional hazard regressions on time to have diabetes. Macrosomia and gestational diabetes were time-dependent variables and time was defined by the self-reported retrospective variables age at having gestational diabetes and a macrosomia infant. As a sensitivity analysis, to determine the association between macrosomia and diabetes independently of GDM, we conducted a multivariable logistic regression and Cox proportional hazard regression with participants without GDM. All analyses were performed in R version 3.5.1 in the survey package, adjusting for the NHANES complex sampling design. *P* ≤ 0.05 was considered statistically significant.

## Results

The comprised 10-year data yielded 10,089 women who answered the NHANES question**,** “Did you have a baby weighting > 9lbs?” and delivered at least one baby. Table 1 summarizes the characteristics of the study sample. The weighted mean age at the time of the survey collection was 51.7 years. Race/ethnicity was distributed as 67.6% White, followed by 12.1% Black, 8.3% Mexican Hispanic, and 5.6% Other Hispanic. About 17% of women had macrosomia and 11.2% had type 2 diabetes.

**Table 1:** Baseline characteristics and bivariate association with macrosomia

Variable	Total ***n*** = 10,089 N (Weighted %)	Macrosomia		***P***-value
		Yes ***n*** = 1767 (17.2%) Weighted %	No ***n*** = 8322 (82.8%) Weighted %	
Age (years), Weighted Mean (SE)	51.7 (0.2)	54.4 (0.4)	51.1 (0.3)	< 0.001
20–44	3514 (35.5%)	29.7%	36.6%	
45–64	3800 (40.4%)	40.0%	40.0%	
≥ 65	2775 (24.1%)	23.4%	29.7%	
Age at time of Macrosomia (years),^a^ Weighted Mean (SE)	26.2 (0.2)	26.2 (0.2)	NA	NA
Gestational Diabetes				< 0.001
No	9295 (92.4%)	88.0%	93.3%	
Yes	775 (7.6%)	12.0%	6.7%	
Age at time of Gestational Diabetes (years),^b^ Weighted Mean (SE)	28.2 (0.3)	28.2 (0.6)	28.2 (0.3)	1.000
Parity				< 0.001
Primiparous	1958 (21.7%)	11.2%	23.9%	
Multiparous	8131 (78.3%)	88.8%	76.1%	
Obesity				< 0.001
Underweight/Normal	2686 (30.0%)	22.4%	31.6%	
Overweight	2937 (29.5%)	29.0%	29.7%	
Obese	4367 (40.5%)	48.7%	38.7%	
Race/Ethnicity				< 0.001
Non-Hispanic White	4201 (67.7%)	70.8%	67.0%	
Non-Hispanic Black	2164 (12.1%)	9.8%	12.5%	
Mexican American	1656 (8.3%)	8.8%	8.2%	
Other Hispanic	1194 (5.6%)	6.5%	5.4%	
Other Race, Including Multiracial	874 (6.4%)	4.0%	6.8%	
Diabetes				< 0.001
No	7234 (76.4%)	70.2%	77.7%	
Yes	2855 (23.6%)	29.8%	22.3%	

Rao-Scott chi-square test and two sample t test were used to evaluate bivariate association with macrosomia, adjusting for the complex sampling design

*N* Unweighted frequency, *Weighted %* Weighted column percentage, *SE* Standard error

^a^Among the women who had macrosomia

^b^Among the women who had gestational diabetes

Table 1 also displays the bivariate association between demographic characteristics and history of macrosomia. All demographic variables were significantly associated with macrosomia except age at time of GDM diagnosis. In the bivariate analysis, macrosomia was associated with a significantly increased risk of maternal type 2 diabetes later in life (Table 2). Among women with diabetes, 21.7% had macrosomia in their pregnancy, which was higher compared to 15.8% the rate of macrosomia among women without diabetes. The other variables of age, GDM, parity, obesity, and race/ethnicity were also significantly associated with diabetes. In the multivariable logistic regression model, macrosomia, age, GDM, race/ethnicity, and obesity were significant (Table 3). Even while accounting for the demographic factors, a history of having a macrosomic infant showed a weak but independent association with future development of type 2 diabetes. The odds of having type 2 diabetes among women with a history of macrosomia was 21% higher compared to the counterparts. Other known risk factors like older age, GDM, obesity and non-white minority racial/ethnic groups were also associated with an increased odds of type 2 diabetes in the model, as expected. Mexican Hispanic has the highest odds of diabetes among all the other racial/ethnic groups compared to whites (odds ratio [OR] = 1.86), followed by ORs of 1.71 Others, 1.51 Other Hispanics, and 1.33 Non-Hispanic Black.

**Table 2:** Bivariate Association between Baseline Characteristics and Diabetes

Variable	Diabetes		***P***-value
	Yes Weighted %	No Weighted %	
Age (years), Weighted Mean (SE)	59.2 (0.3)	49.4 (0.3)	< 0.001
20–44	16.5%	41.3%	
45–64	44.0%	39.2%	
≥ 65	39.2%	19.5%	
Macrosomia			< 0.001
No	78.3%	84.2%	
Yes	21.7%	15.8%	
Age at time of Macrosomia (years),^a^ Weighted Mean (SE)	26.0 (0.3)	26.4 (0.2)	0.367
Gestational Diabetes			< 0.001
No	87.7%	93.8%	
Yes	12.3%	6.2%	
Age at time of Gestational Diabetes (years),^b^ Weighted Mean (SE)	28.3 (0.3)	28.2 (0.4)	0.910
Parity			< 0.001
Primiparous	17.3%	23.1%	
Multiparous	82.7%	76.9%	
Obesity			< 0.001
Underweight/Normal	16.5%	34.2%	
Overweight	25.5%	30.8%	
Obese	58.1%	35.0%	
Race/Ethnicity			0.002
Non-Hispanic White	64.2%	68.8%	
Mexican American	13.4%	11.7%	
Other Hispanic	9.9%	7.8%	
Non-Hispanic Black	6.0%	5.4%	
Other Race, Including Multiracial	6.6%	6.3%	

Rao-Scott chi-square test and two sample t test were used to evaluate bivariate association with macrosomia, adjusting for the complex sampling design

*Weighted %* Weighted column percentage, *SE* Standard error

^a^Among the women who had macrosomia

^b^Among the women who had gestational diabetes

**Table 3:** Associated odds ratios and 95% confidence intervals for univariable and multivariable logistic regression models predicting diabetes

Variable	Crude OR (95% CI)	AOR (95% CI)
Macrosomia	1.48 (1.28–1.71)***	1.21 (1.00–1.45)*
Age		
20–44 years	1.00	1.00
45–64 years	2.81 (2.44–3.24)***	3.26 (2.78–3.82)***
≥ 65 years	5.03 (4.29–5.89)***	7.31 (6.12–8.73)***
Race/Ethnicity		
Non-Hispanic White	1.00	1.00
Non-Hispanic Black	1.23 (1.06–1.43)**	1.33 (1.12–1.57)**
Mexican	1.35 (1.14–1.60)***	1.86 (1.57–2.20)***
Other Hispanic	1.18 (1.02–1.37)*	1.51 (1.27–1.80)***
Other	1.13 (0.89–1.42)	1.71 (1.33–2.20)***
Obesity		
Underweight/Normal	1.00	1.00
Overweight	1.72 (1.45–2.03)***	1.62 (1.35–1.95)***
Obese	3.44 (2.95–4.01)***	3.46 (2.94–4.07)***
Gestational Diabetes	2.11 (1.73–2.58)***	2.77 (2.22–3.46)***
Parity		
Primiparous	1.00	1.00
Multiparous	1.44 (1.20–1.72)***	0.98 (0.80–1.19)

Logistic regressions were conducted adjusting for the complex sampling design

*OR* Odds Ratio, *CI* Confidence Interval, *AOR* Adjusted odds ratio

*P ≤ 0.05. **P < 0.01. ***P < 0.001

The results from Cox proportional hazard regressions are provided in Table 4. Similar to the logistic regressions, we found significant association between diabetes and macrosomia, GDM, race/ethnicity, and obesity. The expected hazards of having type 2 diabetes is 1.66 times higher in a woman with a history of macrosomia compared to the counterparts as 1 year increases in age. Mexican Hispanic women had the highest hazards of diabetes among all the other racial/ethnic groups compared to whites (hazard ratio [HR] = 2.18), followed by HRs of 1.83 Others, 1.66 Other Hispanics, and 1.57 Non-Hispanic Black as 1 year increases in age.

**Table 4:** Associated hazard ratios and 95% confidence intervals for univariable and multivariable Cox Proportional Regression models predicting diabetes

Variable	Crude HR (95% CI)	AHR (95% CI)
Macrosomia	1.95 (1.74–2.19)***	1.66 (1.46–1.89)***
Race/Ethnicity		
Non-Hispanic White	1.00	1.00
Non-Hispanic Black	1.81 (1.61–2.04)***	1.57 (1.40–1.76)***
Mexican	2.61 (2.29–2.97)***	2.18 (1.94–2.44)***
Other Hispanic	1.91 (1.64–2.21)***	1.66 (1.43–1.92)***
Other	1.57 (1.30–1.91)***	1.83 (1.50–2.23)***
Obesity		
Underweight/Normal	1.00	1.00
Overweight	1.57 (1.33–1.86)***	1.49 (1.26–1.75)***
Obese	3.48 (2.99–4.06)***	2.94 (2.51–3.44)***
Gestational Diabetes	5.52 (4.82–6.33)***	4.34 (3.75–5.01)***
Parity		
Primiparous	1.00	1.00
Multiparous	0.92 (0.78–1.10)	0.79 (0.68–0.93)**

*HR* Hazard Ratio, *CI* Confidence Interval, *AHR* Adjusted hazard ratio

**P < 0.01. ***P < 0.001. Cox proportional regression was conducted adjusting for the complex sampling design. Macrosomia and gestational diabetes are time-dependent variables

Table 5 presents the sensitivity analysis results including only women who did not have history of GDM. The results were comparable to those in multivariable logistic and Cox proportional hazard regressions. Macrosomia was significant risk factor for having diabetes (OR = 1.24, HR = 1.83).

**Table 5:** Sensitivity analysis result for participants who did not have gestational diabetes

Variable	AOR (95% CI)	AHR (95% CI)
Macrosomia	1.24 (1.03–1.49)*	1.83 (1.63–2.07)*
Age		
20–44 years	1.00	
45–64 years	3.63 (3.09–4.27)***	
≥ 65 years	7.89 (6.60–9.42)***	
Race/Ethnicity		
Non-Hispanic White	1.00	1.00
Non-Hispanic Black	1.29 (1.09–1.52)**	1.53 (1.36–1.73)***
Mexican	1.88 (1.56–2.26)***	2.28 (2.00–2.60)***
Other Hispanic	1.43 (1.36–1.72)***	1.72 (1.49–1.98)***
Other	1.76 (1.36–2.28)***	1.90 (1.60–2.26)***
Obesity		
Underweight/Normal	1.00	1.00
Overweight	1.69 (1.40–2.04)***	1.49 (1.27–1.74)***
Obese	3.50 (2.96–4.13)***	3.16 (2.71–3.69)***
Parity		
Primiparous	1.00	1.00
Multiparous	0.93 (0.76–1.14)	0.79 (0.67–0.92)**

*AOR* Adjusted odds ratio, *AHR* Adjusted Hazard Ratio, *CI* Confidence Interval

*P ≤ 0.05. **P < 0.01. ***P < 0.001. Multivariable logistic regression and Cox proportional hazard regression were conducted adjusting for the complex sampling design. In the Cox proportional regression, macrosomia and gestational diabetes were time-dependent variables

## Discussion

We utilized nationally representative data to assess the effect of history of macrosomia on diabetes and found that a macrosomic infant alone is an independent marker for developing type 2 diabetes later in life even after adjusting for GDM and other sociodemographic factors. Societies recommendations from the ADA and AACE recommend screening women for type 2 diabetes if they have a history of GDM or gave birth to an macrosomic infant. The theory is that an macrosomic infant is a marker for having a pregnancy affected by GDM. Often, however, a patient may not remember if they had GDM or may not have been tested, and thus asking the birthweight of their neonate is a surrogate used to assess whether or not they had impaired glucose tolerance during pregnancy [25]. The United States Preventative Services Task Force (USPSTF) only mentions screening those affected by GDM and not those with an macrosomic infant alone [26], as does the American College of Obstetricians and Gynecologists (ACOG), who recommend screening women with GDM for type 2 diabetes or impaired glucose tolerance 6–12 weeks postpartum and then every 1–3 years afterward [27]. Overall implementation of these screening guidelines range from 20 to 54% [28].

Pregnancy is a window into future health, and the postpartum period and reproductive years are important times to initiate regular screening for cardiometabolic health [29]. Such screening identifies women earlier who will benefit from lifestyle interventions. GDM is a well-documented risk factor for development of type 2 diabetes later in life [2]. The impaired glucose intolerance during pregnancy is likely associated with genetic susceptibility and behavioral risk factors that lead to impaired insulin secretion and utilization at an older age. The correlation could also be causational, with a theory that GDM stimulates earlier pancreatic beta cell dysfunction.

While GDM is a known risk factor for developing type 2 diabetes, few studies have looked at the long-term impacts for women of giving birth to an LGA infant without concomitantly diagnosed GDM. Researchers in Finland followed approximately 800 women after pregnancies affected and unaffected by GDM. After an average of 7 years from the index pregnancy, women with an LGA infant and no diagnosis of GDM did not have higher rates of type 2 diabetes or metabolic syndrome compared to controls [30, 31]. A similar study in Iran followed 570 women 9 years after a pregnancy unaffected by GDM who delivered an LGA infant, and compared them to 628 women with appropriately grown infants. Giving birth to a larger infant did not predispose them to higher rates of type 2 diabetes or hypertension during the follow-up period, while adjusting for maternal age and BMI [32]. Finally, Moses and colleagues in Australia evaluated 36 women with appropriate for gestational age (AGA) and LGA infants 2 years after a pregnancy unaffected by GDM. They found no biochemical differences in terms of abnormal glucose of lipid profiles between the two groups [33].

Our study is unique in evaluating a large sample size of women in the United States with greater than 20 years of follow-up. Unlike the smaller studies with shorter follow-up time performed outside the U.S., our analysis demonstrated a 20% increased risk of future development of type 2 diabetes compared to counterparts who did not have a pregnancy affected by GDM, independent from other known risk factors such as race and BMI. Although the effect size is small, our findings provide support to the screening recommendations offered by the ADA and AACE.

Further studies are needed to understand the etiology of this relationship. One such hypothesis is that maternal hyperlipidemia leads to increased birth weight, even in the absence of maternal hyperglycemia. Wang et al. demonstrated that maternal serum lipid content during the third trimester was proportionally related to macrosomia risk in women without diabetes [34]. Another study investigated the impact of lipid concentrations in women with Non-Alcoholic Fatty Liver Disease (NAFLD), and also demonstrated a proportional relationship to birthweight, with triglycerides contributing to most of this risk [35].. Thus, maternal hyperlipidemia may be a contributing factor to macrosomic offspring, and this risk factor is also known to be associated with development of diabetes mellitus later in life. The proposed mechanisms is via the breakdown of triglycerides, leading to inflammation through the creation of free fatty acids. These substrates cause insulin resistance and β-cell dysfunction by disrupting insulin receptors and glucose transporters [36, 37]. Therefore, the same hypertriglyceridemia that contributes to macrosomia during reproductive years may also induce subclinical inflammation causing β-cell dysfunction and subsequently the development of diabetes mellitus. This study was not designed to investigate this association and further studies should continue to probe not only the risk factors, but also preventative measures for these observed relationships.

Other limitations to this study are inherent with survey-based, cross-sectional studies. The database is self-reported information and subject to recall bias, as the mean age of respondents was 22 years older than when they had a pregnancy affected by macrosomia. Universal screening for GDM was just starting to be widely adopted at that time, and thus there may have been women who were not screened for GDM due to not having risk factors for the condition. We only included variables available in the NHANES database. Delivery of a macrosomic infant and parity may be associated, but we were unable to delineate which birth in multiparous women was affected by macrosomia. Additionally, there was no information regarding the mother’s body mass index during pregnancy, gestational weight gain, or fetal distribution of fat mass which are other important confounding variables for development of impaired glucose tolerance and excessive hyperglycemia during pregnancy. The NHANES database does not include actual birthweight data linked mother-baby dyads, so we are unable to assess the association of all LGA offspring or the magnitude of birthweight and type 2 diabetes development. However, 9lbs or 4082 g is LGA for neonates born at 40 weeks gestation [7], and thus is a representative and more sensitive surrogate marker for the pathophysiology of elevated birthweight.

The data highlights the importance of obtaining an obstetric history during a preventative health visit as well as continuing to improve screening for women in the postpartum period and throughout reproductive years. The correlation of type 2 diabetes and delivery of an macrosomic infant is not as strong as that of obesity or GDM, but appears to have a moderate contribution that warrants extra counseling. Providing education and screening for women who gave birth to an macrosomic infant could decrease progression of developing type 2 diabetes in the future by prompting early lifestyle interventions. This information can also help guide and motivate women to maintain appropriate fetal growth in subsequent pregnancies.

## Conclusion

Pregnancy affected by macrosomia is correlated with future development of type 2 diabetes. Women who gave birth to an infant larger than 9 lbs. in the absence of GDM should still be counseled on the risk of acquiring type 2 diabetes and offered earlier screening for this condition.

## Data Availability

The NHANES data is publicly available dataset that can be accessed at https://wwwn.cdc.gov/nchs/nhanes/Default.aspx
